# Recommendations for reporting results of diagnostic genomic testing

**DOI:** 10.1038/s41431-022-01091-0

**Published:** 2022-04-01

**Authors:** Zandra C. Deans, Joo Wook Ahn, Isabel M. Carreira, Elisabeth Dequeker, Mick Henderson, Luca Lovrecic, Katrin Õunap, Melody Tabiner, Rebecca Treacy, Christi J. van Asperen

**Affiliations:** 1 GenQA, Laboratory Medicine, Royal Infirmary of Edinburgh, Little France Crescent, Edinburgh, EH16 4SA UK; 2 Cambridge University Hospitals Genomic Laboratory, Cambridge University Hospitals NHS Foundation Trust, Cambridge Biomedical Campus, Cambridge, UK; 3 University of Coimbra, Faculty of Medicine, Cytogenetics and Genomics Laboratory, iCBR/CIMAGO, Center for Innovative Biomedicine and Biotechnology, Coimbra, Portugal; 4 University of Leuven, Department of Public Health and Primary Care, Biomedical Quality Assurance Research Unit, Leuven, Belgium; 5 ERNDIM, Biochemical Genetics, Specialist Laboratory Medicine, St James University Hospital, Leeds, UK; 6 Clinical Institute of Genomic Medicine, University Medical Centre Ljubljana, Ljubljana, Slovenia; 7 Department of Clinical Genetics, United Laboratories, Tartu University Hospital, Tartu, Estonia; 8 Institute of Clinical Medicine, University of Tartu, Tartu, Estonia; 9 GenQA, John Radcliffe Hospital, Oxford University Hospitals NHS Foundation Trust, Headley Way, Oxford, OX3 9DU United Kingdom; 10 Department of Clinical Genetics, Leiden University Medical Center, Leiden, the Netherlands

**Keywords:** Diseases, Genetics

## Abstract

Results of clinical genomic testing must be reported in a clear, concise format to ensure they are understandable and interpretable. It is important laboratories are aware of the information which is essential to make sure the results are not open to misinterpretation. As genomic testing has continued to evolve over the past decade, the European Society of Human Genetics (ESHG) recommendations for reporting results of diagnostic genetic testing (biochemical, cytogenetic and molecular genetic) published in 2014 have been reviewed and updated to provide the genomic community with guidance on reporting unambiguous results.

## Introduction

Genetic testing is an essential tool to identify rare inherited and complex disease and it is continually evolving as new technologies are implemented into diagnostic laboratories. It is often expected to gain as much information from the genome as possible to increase the chance of a diagnosis. Improved diagnostic yield provides opportunities for informed life choices but it impacts not just on the individual but also wider family members to identify potentially high-risk individuals for disease susceptibility. A firm diagnosis is not always possible but a wealth of genomic data can be utilised to exclude certain diagnoses. The understanding of these genomic data, which can often be challenging, the clinical details, limitations of the testing performed and the clear report of what has and has not been detected is critical for ensuring the accurate interpretation of the results obtained.

The requirement in many countries for medical laboratories to be accredited to ISO 15189:2012 [1] has aided the standardisation of the reporting of genomic results. However, results of external quality assessments highlight the need for laboratories to frequently review the format of reporting results, as standards and methodologies improve, new technologies are introduced and experience with clinical interpretation grows.

In light with the changes which have occurred in genetic testing since 2014, the European Society of Human Genetics (ESHG) recommendations for reporting results of diagnostic genetic testing (biochemical, cytogenetic and molecular genetic) [2] have been reviewed and updated to provide the genomic community with guidance on approaches to reporting clear and unambiguous results in the current landscape. All sections were reviewed and amended where required and the key areas of change are summarised in Table 1.

**Table 1:** Summary of updates to the previous iteration of the European Society of Human Genetics (ESHG) recommendations for reporting results of diagnostic genetic testing (biochemical, cytogenetic and molecular genetic) [2].

Section	Summary
Clinical information accompanying the samples	The minimal set of clinical information accompanying the samples has been removed. Each laboratory has their local requirements for sample acceptance so the authors agreed that this document should focus on the information required for the report rather than sample receipt. The relevant details required to be present on the report are summarised.
Contents of the report • Administrative • Identification • Clinical question • Test specifications	The contents of the report section has been updated to align with ISO15189 for ease of use. The increased use of electronic reporting has been addressed. Information on the importance of understanding and reporting the limitations of the test performed in the context of current methodologies has been provided. The use of standardised nomenclature, legacy nomenclature is discussed. Guidance on reporting tandem repeat expansions is given.
Contents of the report • Interpretation of the results	The reporting of variant pathogenicity classification is discussed. Guidance on the reporting of no clinically significant findings, incidental findings and recommendations for further testing has been included.
Reporting results from testing of multiple individuals	This section has been updated in line with current GDPR.
Amended reports	New section added.
Sample storage reports	New section added.

## Scope of document

This document provides recommendations on the appropriate reporting of genomic test results for inherited disorders in the postnatal setting by biochemical genetic, cytogenomic and molecular genetic techniques. The focus is on the clear, accurate and concise reporting of results. Laboratory testing, application of variant classification systems and guidance on reanalysis are not within the scope of this document.

It is acknowledged that there are national recommendations available in some countries and this guidance is intended to compliment those already in use. It is recommended that current specific guidance on reporting genomic tests with specific requirements, such as preimplantation genetic diagnosis, non-invasive prenatal testing, biochemical newborn screening, polygenic risk scores, acquired disorders and pharmacogenetics is followed. However, the basic principles of reporting genetic results outlined in this document can be followed. Consent should be sought, where required, in line with local regulations.

## Definition of report

As stated in the previous iteration of the ESHG recommendations for reporting results of diagnostic genetic testing (biochemical, cytogenetic and molecular genetic) [2], reports are specific formal medical documents (including electronic versions) from the laboratory to the referring physician and/or other healthcare professionals regarding the results and interpretation of genetic testing in a patient and/or members of a family. The main goal of reports is to provide a clear, concise, accurate, fully interpretative and authoritative answer to a clinical question.

## Contents of the report

Medical genetics is still rapidly evolving, for patient safety it is considered essential that all analytical results from genomic testing are adequately interpreted and communicated to the referring clinicians in a comprehensive way. A patient report is read and discussed by many different parties within and outside the hospital, including patients themselves. The elements in the report are used for diagnosis, prognosis or to gain information on important possible predictive factors for various decisions. With the implementation of genomic testing, for example whole exome and genome sequencing, where a large amount of information is generated, many laboratories struggle on how elaborate the clinical report must be therefore the need for adequate reporting guidelines is growing. Recently, the focus on structured and synoptic reporting is growing with laboratories and clinicians alike striving for standardisation of reports. A reason that succinct reports must be considered is the growing volume of data generated in laboratories that makes it no longer feasible to format the report in a narrative way. Concise reports can help clinical scientists to focus on the data that are necessary for a complete report. This will enhance the quality of the report and lower the risk that important information is lacking [3]. Guidelines and recommendations for the interpretation of data and subsequent reporting in medical laboratories, especially for hereditary diseases exist [1, 4–8] should be taken into account in conjunction with this guidance. The following elements should be present in every report.

### Administrative

A clear, unambiguous identification of the examination is needed, this could be the title of the report but not necessarily.

The identity of the laboratory performing the analysis and issuing the report, with full contact details (including phone number). If parts of analyses have been carried out in other laboratories, this fact must be clearly and unequivocally stated [1].

Full date of when the report is authorised.

Electronic reports may be issued but page numbers indicating the total number of pages must be included when reports can be printed. This is essential when multiple pages are used (e.g. 1/1 or page 1 of 2) and unique patient identification must be provided on each page (see Patient identification section).

Name and full address (including phone number) of the requester / the physician referring the patient.

The report must be electronically or manually signed by the authorized specialist individual who validated the analysis and interpreted the result; co-validation and co-signature by a second competent person is recommended (and mandatory in some countries). The name and function of signatories must be given.

In order to avoid errors and/or misinterpretation, transcription of all or part of this report is inadvisable. It is recommended to add a standard phrase indicating that “this report may not be copied or reproduced, except in totality”.

### Patient identification

There must be clear identification of the patient since this is a crucial element through the whole process of care delivery. As a minimum, this should include:

- Full given name(s)
- Surname
- Unequivocal date of birth and/or personal identification code

The gender of the patient should be stated.

Foetal samples (chorionic villi samples, amniocytes etc.) should be clearly identifiable and reported as unique individuals, with reference to the mother’s identification. The date of sample may be used to identify separate pregnancies and separate samples from the same foetus.

Where legislation (e.g. data protection legislation) prohibits the transmission of identifiable personal information, a code may be used instead of the patient’s name.

Ethnic/geographic origin, if relevant.

Patient identifications should be included on each page of a multi-page report.

### Sample identification

There must be clear identification of the sample being tested as this is a crucial element through the whole process of care delivery.

The date of primary sample collection, (and time, when available; this information is crucial for biochemical genetic testing) must be stated.

Type of primary sample and information on the status of the sample if relevant (e.g. frozen, decomposed, haemolyzed).

Date (and time) of sample arrival to the laboratory (and where necessary comments regarding sample suitability with respect to acceptance / rejection criteria).

Material that has been tested (e.g. “EDTA blood”, “cultured amniocytes”, “DNA extracted from…”, “heparin plasma”, “urine” etc.). When the sample has been processed in another laboratory e.g. DNA has been extracted, plasma has been separated and frozen, amniocytes have been cultivated etc.) then this should be stated.

Unique identification number for each sample tested.

### Restatement of the clinical question

The interpretation of genetic and genomic test results depends usually on the clinical context and history of the patient. Based on the information, provided by the attending physician or genetic counsellor, the reports of genetic testing should explicitly restate the clinical question being asked. This usually comprises at least the following three elements:

- The disease(s) or marker(s) being requested for analysis (e.g. cystic fibrosis, lysosomal storage disorders, inborn errors of metabolism, chromosomal abnormalities);
- The type of required testing (e.g. diagnostic confirmation, carrier status, prenatal diagnosis);
- The referral reason i.e. why the request is being made (e.g. previous family history, multiple congenital abnormalities, fasting hypoglycaemia).

When the referral is due to abnormal results of previous genetic testing in either the same laboratory or a different laboratory this fact must be clearly indicated in the report, with a reference to the relevant previous report.

### Specification of tests performed

Provide concise information on the method(s) used to determine the results reported. Additional information may be in footnotes, appendices or available on a document-controlled website.

Give full details of the extent of the tests i.e. the scope of the test (e.g. which exons in which transcript screened for pathogenic variants, which fluorescence in situ hybridisation (FISH) probes used, specific variants detected by the assay, microarray platform design details). This information is particularly important when reporting no abnormality detected results.

The technical sensitivity and practical resolution of the test must be provided where applicable and based on internal data and validation testing. This could be described with a concise statement about the limitations (e.g. the banding resolution of cytogenetic analyses, microarray average resolution, threshold for detection of mosaicism) or by stating the commercially available kit or software package is used (including the manufacturer, reference and version of the kit) and, where applicable, the list of variants included in the version used [6]. Where appropriate, give the detection rate (sensitivity) in the population of origin of the individual tested.

If the testing performed is incomplete or fails to achieve the minimum quality required, details and further recommendations should be given. When appropriate, the clinician should be invited to send a repeat sample.

### Reporting the results

The results must be presented in a brief and unambiguous form. If several different tests have been performed, the results should be shown separately for each of the tests. The terms “positive” and “negative” can be ambiguous and should not be used.

The report should be tailored to the type of referral, for example:

- Diagnostic (including trios)
- Predictive/Presymptomatic/Carrier
- Prenatal
- Population testing

Gene reference sequence and/or genome build and nomenclature of detected genetic variants should be meaningful, unambiguous and consistent using the recommended Human Genome Variation Society (HGVS) nomenclature [9] valid at the time of reporting. Since both HGVS nomenclature and the reference sequences change in time, other descriptions of the variant that have been widely used in the past may be given in parentheses, if appropriate. Sequence-based nomenclature for the description of large structural variation and large copy number variation is provided in the International System for Human Cytogenomics Nomenclature (ISCN) [10].

Referenced legacy or historical exon numbering or variant nomenclature may be included in the report if appropriate.

For tandem repeat disorders, reference ranges should be provided for the normal, intermediate and disease-causing ranges. The source of these ranges should be stated.

If more than one possibly pathogenic variant has been detected, a comment on the phase of variants (which may be unknown) should be added taking into account the expected mode of inheritance of the disorder.

For cytogenomic testing, region specific assays and microarray testing, the most recent ISCN nomenclature should be used. ISCN for FISH investigations is not mandatory; results should otherwise be described as simply and clearly as possible, both in the headline summary and in the report text.

It may be useful to give a list of genes involved (by Human Genome Organisation Gene Nomenclature Committee (HGNC) [11] symbol and/or Online Mendelian Inheritance in man (OMIM) [12] number) when reporting copy number variants and for other tests involving multiple genes. This information may be provided as an appendix or supplied upon request so as not to distract from the clinical content of the report.

Include details of diagnosis or associated syndrome/disease.

For biochemical genetic testing using qualitative tests, the report should clearly distinguish normal results from non-specific findings and from clearly abnormal results (including an indication of which diseases are most likely). For quantitative analyses of single analytes, both the value and the reference range should be given. For enzyme assays it is usual to show, in addition, the activities of controls run in parallel with the patient sample; reports on enzyme activity should contain the enzyme commission number of the enzyme. For quantitative profile analyses, the report should only summarize the major findings distinguishing normal, non-specific and disease-specific profiles; numeric values of the profile analyses should be sent as attachments.

### Interpretation of the results

Differences exist between countries as to where the boundary of the responsibilities of laboratory specialist versus the clinician lies therefore laboratory and clinical specialists should discuss and document what the minimal interpretation in the laboratory report should be.

The report should at least contain all relevant information used to interpret the result e.g. literature resources, reference sequences, databases of normal variation like the Genome Aggregation Database (GnomAD) [13], Database of Genomic Variants (DGV) [14], or disease-specific database like the Leiden Open Variation Database (LOVD) [15].

It is mandatory to suggest further family studies where these would give additional information for the proband, therefore this should be clearly stated on the report.

The results must be viewed as interpreted based on current knowledge and thus may change in the future due to additional evidence.

The report must provide a full and clear interpretation of results depending both on the clinical context and the reason for referral (e.g. diagnostic test, carrier test, prenatal test). Reports on patients may be read by a variety of professionals involved in their care, many of whom will be unable to interpret fully the results of genetic testing. In order to provide a full interpretation, the results must be viewed in the context of the relevant clinical and family information available (e.g. relationship between the patient and the index case where relevant, family pedigree, ethnic background, clinical and laboratory data provided by the referring physician and others).

If more tests have been performed using the same sample(s), the interpretation part of the report should integrate the individual results and should answer the clinical question in a clear and concise way. This integration of findings with relevance to the tested individual is especially important when multiple susceptibility genetic variants have been analysed.

It is recommended that the report highlights a conclusion (bold, underline, large font, text in a box etc.). Significance of results in respect to the referral reason (clinical question) should be clearly stated, be classified according to current sequence variant guidelines and belong to one the following five categories [4]. In some cases there may be more than one conclusion:

- Pathogenic (disease specific, pathological) finding(s): Findings that are outside of the physiological variation and that are at the present state of knowledge unequivocally associated with a clinically relevant disease or group of diseases. Pathogenicity of findings should be clearly supported from data in literature/databases, segregation analysis, functional tests of genetic variants and other relevant means.
- Likely pathogenic findings: this category is distinguished from clearly pathogenic findings according to calculating metrics within specific guidelines [4, 16]. Overall, these variants have strong characteristics and supporting evidence to suggest that they are likely disease causing either by type of variant (partial gene deletion in established haploinsufficiency gene, but not previously described; variant leading to amino acid change at the same codon as known pathogenic variants have been described). When evaluating the evidence, care must be taken to ensure there is no duplication.
- Finding(s) of uncertain significance: are also classified according to calculating metrics within specific guidelines (same as above). These might be findings outside of the physiological variation but with possible or putative relevance to the clinical question asked (i.e. clinical context) e.g. novel missense variants, novel putative splicing variants with unproven effect at RNA level, mosaicism or novel rearrangements in cytogenomic analyses, novel or rarely reported copy number variations, intermediate analyte concentration/enzyme activity suggesting either heterozygosity or a mild phenotype. There is country specific variation on whether VUS are reported. It is important that they are recorded within the laboratory to enable future review, however some variants with a highly likelihood of affecting clinical management should be reported.

- Benign and likely benign variant(s), when no clinically significant finding(s) is detected, see Reporting of no clinically significant findings section.

When appropriate to the clinical referral, genetic carrier risks should be stated. Risk estimates may require the application of Bayesian calculations.

Indicate if further tests could be undertaken to improve the accuracy or scope of the interpretation or classification of the pathogenicity of the variant. This may include tests for additional disorders or additional tests to more fully investigate the variant in question. If the additional tests suggested are not performed “in-house”, alternative specialist laboratories where the sample may be sent may be proposed.

Suggest any other information which could be supplied or obtained by the referring clinician which might improve the accuracy of your interpretation (e.g. arranging testing of the index case in a family to confirm a diagnosis or to determine which variants are present).

The report should carry the reminder to the referring physician that “genetic tests should be accompanied by genetic counselling” (or similar).

When a new diagnosis is made (confirmatory result in a diagnostic confirmation) it should be clear from the report that the result has “potentially important implications for other family members” or equivalent. Depending on the context, it may be appropriate to explicitly mention the recommendation to test the partner, the possibility of cascade screening tests of at-risk relatives, potential clinical review of at-risk relatives, the possibility of prenatal diagnosis or preimplantation genetic diagnosis. Where appropriate, the risk for future offspring should be calculated and provided (using carrier frequencies from an appropriate population).

Description of genotype can include the correct assignment of phase, which may require the study of familial segregation of alleles. When appropriate, reports must explicitly mention that “both parents should be tested to confirm their carrier status and to provide formal confirmation of the diagnosis of their child”.

Prenatal or pre-symptomatic diagnosis must be offered when appropriate. The report should not state that this is “indicated” or “necessary” as this decision would be made out-with the laboratory

In summary, as many details are required, it is often requested to provide a short summary of the test results and interpretation, and to include all the additional details as a supplementary document to the report [4, 16]. When no summary page is provided, the clinically critical data should be present on the first page of the report [17, 18].

### Reporting of no clinically significant findings

When no clinically significant finding(s) is detected then this must be reported.

To avoid possible misinterpretation of the results, benign and likely benign variant(s) should not be included in the report (when compatible with existing disease or national reporting guidelines) [10, 17]. Findings which have no clinical significance, are within the range of physiological variation for the given individual considering ethnicity, age, sex, maturity and other relevant factors; e.g. nucleic acid sequence corresponding to the reference sequence or containing genetic variants considered usual variations at the time of reporting, concentrations of analytes in quantitative biochemical genetic tests within reference ranges, results of qualitative biochemical genetic tests showing a usual result/profile.

The report must clearly state what has been tested and any limitations which should be taken into account for clinical management, see section 4.5.

It is important to state that no clinically significant variants have been identified rather than stating that the patient is ‘normal’ or ‘negative’.

The report should state that further appropriate investigations may be considered e.g. further testing, testing of alternative sample types, further clinical evaluation, family studies etc.

When reporting biochemical genetic tests where results show nothing abnormal it is important to report the results of all tests or test groups performed. A global statement such as ‘metabolic testing showed no abnormality’ is unhelpful because the clinical record must be able to evidence which categories of disorder have been potentially excluded. It may also be relevant in some circumstances to emphasise that failure to detect abnormal metabolites will not exclude the diagnosis of disorders where metabolite excretion is intermittent and varies according to clinical circumstance e.g. stress or fasting. This is also relevant for those disorders where some variants are low excretors e.g. glutaric aciduria type1 [19].

### Incidental findings

Incidental finding(s) with possible clinical relevance are findings indicating a clinically relevant issue but unrelated to the clinical question that was asked (e.g. signs of sex chromosome abnormality when analysing an X-linked disorder or evidence of predisposition to an unrelated condition). The decision on whether to report such findings should follow national policy and will depend on how the patient has been counselled about this possibility. A clear policy on reporting incidental findings should be in place in all institutions offering genetic testing.

If policy is to report incidental findings, then only pathogenic (class 4/5) variants which would impact on patient management should be reported.

Incidental findings also include the detection of family relationships as not being stated. A clear policy on reporting these results should be in place.

## Interim reports

It may in some circumstances be useful to issue a report before all studies are complete (e.g. when indicative preliminary results have been obtained but a long delay is expected before the final results will be ready or further samples are required to complete the testing or interpretation). Interim reports should be clearly marked as such and should be worded to avoid misinterpretation of their status. Thus, phrases or summary statements appearing to give a definitive result should not be used. It should be clearly stated which analyses are still underway.

The final report should clearly state which are the new results, and should include a general interpretation taking account of all results. This final report should reference the interim report(s) and clearly state that this report supersedes the interim report.

## Reporting results from testing of multiple individuals

As a general rule, each individual should be reported on a separate and unique document, since the reports will ultimately be filed in individual patient or family files (as well as for reasons of confidentiality and compliance with the General Data Protection Regulation [20]). It is recognised that, in some countries, it is not permissible to mention more than one individual in a clinical report however reports should provide sufficient information to enable the physician to interpret the full picture.

When several family members are analysed simultaneously, policies vary as to whether they should be reported on the same or on different reports. This will depend on the disorder, on the nature of the analysis and also on national legislation.

Predictive or pre-symtomatic test results must always be reported as separate, individual reports.

For prenatal diagnosis, it is recommended that the report includes only the result of the foetus. Parental results should be cross-referenced but their results reported separately.

In carrier testing for a recessive disorder for a couple wishing to determine the risk of having an affected child, the test results for one partner must be interpreted in light of the other partner’s results. Laboratories may issue a single report, or separate reports with cross references to the partner’s results (as the couple may separate).

Where the result of the parents is needed to interpret the result in the child only the specific variant detected in the parents is given in the child’s report.

Linked-marker studies are only useful in the context of alleles inherited by several family members, which must be included in the report. However, a single report should provide interpretation and a final risk for each relevant individual in order to answer the clinical question being asked.

## Disclaimers

Disclaimers should only be included where they are relevant and useful.

Where relevant, the report should mention the possibility of errors due to factors beyond the control of the laboratory (e.g. the risk of “non-paternity” and the need for family relationships as stated on the referral forms being correct; limited validity of biochemical testing if pre-analytical conditions were not well controlled).

If appropriate to the case, it may be advisable to state that the “accuracy of the result depends on the clinical information supplied”.

Laboratories may add a note of caution regarding sample identity when reports are based on samples or reports sent from another laboratory.

## Amended reports

If a report is required to be amended then it must be clear that this new report references all previous report(s) issued and clearly states that this report supersedes all others.

The amended report must be issued to all clinicians associated with the original referral or referral laboratory if appropriate.

There must be a policy to ensure that receivers of the amended report are made aware that this is not a copy of the original report but in fact an amended version.

The amended report must highlight what has been changed from previous reports.

The original report must be stored within the laboratory and made available upon request.

## Sample storage reports

Samples may be received which are not for immediate testing. These can be for storage only or DNA/RNA extraction and banking, potentially for later use. A report should be issued as a record of sample storage with details provided on how to initiate a subsequent test request.

When sample stability is limited then this timeframe should be included on the report.
